# Yuan-Hu Zhi Tong Prescription Mitigates Tau Pathology and Alleviates Memory Deficiency in the Preclinical Models of Alzheimer’s Disease

**DOI:** 10.3389/fphar.2020.584770

**Published:** 2020-10-30

**Authors:** A. Iyaswamy, S. K. Krishnamoorthi, Y. W. Liu, J. X. Song, A. K. Kammala, S. G. Sreenivasmurthy, S. Malampati, B. C. K. Tong, K. Selvarasu, K. H. Cheung, J. H. Lu, J. Q. Tan, C. Y. Huang, S. S. K. Durairajan, M. Li

**Affiliations:** ^1^ Mr. & Mrs. Ko Chi-Ming Centre for Parkinson’s Disease Research, School of Chinese Medicine, Hong Kong Baptist University, Hong Kong SAR, China; ^2^ Institute of Biopharmaceutical Sciences, National Yang Ming University, Taipei, Taiwan; ^3^ Medical College of Acupuncture-Moxibustion and Rehabilitation, Guangzhou University of Chinese Medicine, Guangzhou, China; ^4^ Division of Mycobiology and Neurodegenerative Disease Research, Department of Microbiology, School of Life Sciences, Central University of Tamil Nadu, Tiruvarur, India; ^5^ State Key Lab of Quality Research in Chinese Medicine, University of Macau, Macao SAR, China; ^6^ Center for Medical Genetics, School of Life Sciences, Central South University, Changsha, China

**Keywords:** Alzheimer’s disease, P301S tau mice, neurofibrillary tangles, Chinese medicine, yuan-hu zhi tong, microarray, connectivity map

## Abstract

Alzheimer’s disease (AD) is characterized by memory dysfunction, Aβ plaques together with phosphorylated tau-associated neurofibrillary tangles. Unfortunately, the present existing drugs for AD only offer mild symptomatic cure and have more side effects. As such, developments of effective, nontoxic drugs are immediately required for AD therapy. Present study demonstrates a novel role of Chinese medicine prescription Yuan-Hu Zhi Tong (YZT) in treating AD, and it has substantiated the *in vivo* effectiveness of YZT in two different transgenic mice models of AD, namely P301S tau and 3XTg-AD mice. Oral treatment of YZT significantly ameliorates motor dysfunction as well as promotes the clearance of aggregated tau in P301S tau mice. YZT improves the cognitive function and reduces the insoluble tau aggregates in 3XTg-AD mice model. Furthermore, YZT decreases the insoluble AT8 positive neuron load in both P301S tau and 3XTg-AD mice. Using microarray and the “Connectivity Map” analysis, we determined the YZT-induced changes in expression of signaling molecules and revealed the potential mechanism of action of YZT. YZT might regulate ubiquitin proteasomal system for the degradation of tau aggregates. The research results show that YZT is a potential drug candidate for the therapy of tau pathogenesis and memory decline in AD.

## Introduction

Alzheimer disease (AD) is most vulnerable disease affecting the entire elderly population without curable methods. Therefore, there is a serious demand to develop novel and new drugs targeting the vulnerable neurodegenerative disease including AD. Accumulation of insoluble and toxic protein aggregates and phosphorylated tau species is very rarely researched which is the main cause for the neuron loss with cognitive damages in later AD. Even though AD is commonly believed to be a memory disorder, nearly all people identified with AD create neuropsychiatric symptoms (NPS), such as anxiety, motor impairment, hallucinations, and psychosis, at some stages of their disease (Dillon et al., 2013). Recent Aβ-based therapies do not prevent cognitive decline, neurofibrillary tangles (NFT) formation or neurodegeneration (Yoshiyama et al., 2013). Recent research have demonstrated a strong correlation between tau pathology, cognitive decline and NPS in the later part of AD (Geerts et al., 2013).

Presently drugs in market can only mitigate some symptoms but cannot alleviate the disease. The key hallmark features of AD are senile plaques (SP) formation in aggregation of amyloid *β*-peptide (Aβ) and NFTs formation due to tau-aggregation (Hardy, 2006; Wang and Mandelkow, 2016). Other critical early features of AD include an increase in neuroinflammation, decline in synapse number, dysfunction of axonal transport and loss of microtubule (MT) density and stability. In the present market, drug discovery based on reducing Aβ plaques has not shown robust clinical efficacy. In contrast, targeting NFTs seems more likely to be successful because NFTs, which comprises hyperphosphorylated, aggregated, misfolded tau protein, are well correlated with cognitive impairment (Bloom, 2014; Wang and Mandelkow, 2016). Thus, reduction of tau aggregation might be further efficient than Aβ-targeting remedies for AD treatment (Giacobini and Gold, 2013).

The failure of Aβ-based clinical trials challenges the belief that Aβ stimulates tau- arbitrated neurodegeneration. Braak and his associates demonstrated (Braak and Braak, 1995; Braak and Braak, 1996) that tau pathology develops separately from Aβ and tau pathology may possibly the crucial cause for the neurodegeneration in AD. Many studies had supported this hypothesis by finding evidences in AD disease models: Aβ immunotherapy demonstrated a decrease in extracellular SP and intracellular Aβ accumulation and did not mitigate phosphorylated tau pathology in 3XTg-AD (amyloid precursor protein (APP), Presenilin and tau) mice (Oddo et al., 2004). Hence, Aβ focused remedies might be protective in the very initial clinical phases of AD, however when cognitive decline commences with tau pathology, and subsequently tau mitigating drugs may possibly be essential for disease modification.

Traditional Chinese medicine (TCM), is an ancient drug treatment process yet still effective therapeutic approach extensively employed in East Asia, this method holds a greater success for the medication of many neurodegenerative diseases as well as AD for centuries (Geerts et al., 2013). TCM herbs, those showing clinical efficacies, are drawing extensive interest as a source for drug discovering for neurodegenerative diseases including AD. A TCM herbal formula i.e. “Yuan-hu Zhi Tong San” (YZT), is clinically used to treat pain and neuralgia (Yuan et al., 2004); Chinese Clinical Trail Registry (ChiCTR-TRC-10001155). YZT is a relatively simple TCM formula that consists of dried plant material of *Corydalis yanhusuo* (CY) (Y. H. Chou & Chun C. Hsu) W. T. Wang ex Z. Y. Su & C. Y. Wu [Papaveraceae] and *Angelica dahurica* (ADH) (Hoffm.) Benth. & Hook.f. ex Franch. & Sav [Apiaceae], mixed at a ratio of 2:1.

YZT is extensively used for the medication of gastralgia and neuralgia in China (Han and Jiang, 2011). In Australia, YZT capsules are legally allowed to be sold as a pain reliever through the Australian Register of Therapeutic Goods (ARTG-ID-14480). YZT has an array of experimentally proven activities involving anxiolytic, antinociceptive, spasmolytic, anti-inflammatory and vasorelaxant (Xu et al., 2013). Even though NFTs and SP are distinctive indicators of AD, AD may possibly be a multifactorial illness which originated from intricate genetic and environmental risk elements. In terms of how the two herbs interact, YZT extract have been shown to generate synergistic activities on the analgesic impact by enhancing plasma contents of dl-tetrahydropalmatine (Liao et al., 2010). However, the disease-modifying activity of YZT against AD on tauopathies have never been studied in previous studies.

In the present study, we probed whether YZT can improve cognitive memory function and boost the clearance of pathological aggregated insoluble tau in 3XTg-AD and P301S tau mice models. Additionally, we assessed motor function and tau degradative pathway *in vivo* and *in vitro*.

## Materials and Methods

### Quality Analysis of Herbal Materials

Dried herbal materials of *Corydalis yanhusuo* (CY)and *Angelica dahurica* (ADH) were procured from Mr. & Mrs. Chan Hon Yin Chinese Medicine Specialty Clinic in the Hong Kong Baptist University (HKBU) and identified according to the Chinese Pharmacopeia specifications (2010 Edition). The voucher specimens were deposited at the School of Chinese Medicine, HKBU, Hong Kong, China. YZT extract was prepared by mixing dry materials of the plant’s CY and ADH in the ratio of 2:1 and were grinded into powder utilizing a waring mixer. Roughly 1 Kg of powder was immersed in 1 L of 80% alcohol and incubated overnight at room temperature and subsequently obtained extract were steeped. The same process was repeated two times for a complete extraction. Extracted solutions were put together, and around 3–4 L were combined and was condensed under vacuum by rotary evaporation at 50°C. The condensed extract was finally lyophilized (LABCONCO, Laboratory Construction Company, MO, United States) under vacuum of 105 × 10^–3^ µbar. The lyophilized powder from different batches were identified for their purity and then stored at 4°C. The chemical ingredients of every single batch of YZT, CY and ADH were tested for its purity using LC-TOF/MS. A detailed method has been described in our previous publications (Durairajan et al., 2017; Iyaswamy et al., 2020).

### Animals and Drug Treatment

Animal experiments were approved by the Committee on the Use of Human and Animal Subjects in Teaching and Research (HASC approval # HASC/13-14/0165) in HKBU and the Committee on the Use of Live Animals for Teaching and Research (CULATR #3314), at the University of Hong Kong. Animal experiments performed in agreement with the applicable guidelines and procedures of HASC and CULATR. We utilized P301S and 3XTg-AD mice models for assessing the effectiveness of YZT in tau pathology. “Generation of P301S transgenic mice overexpressing the shortest human four-repeat tau isoform (0N4R) under the control of a neuron-specific Thy-1.2 promoter element” has been described previously (Allen et al., 2002). Homozygous P301S tau transgenic and age-matched wild type mice ranging from four to six of weeks age were included in the current study. There were three groups, with N = 14 mice per group in P301S study. In brief, P301S mice were treated every day via food admixture with YZT of 2 or 4 g per kg body weight or vehicle. The study protocol was approved by the HASC of HKBU. Triple transgenic mice (3XTg-AD), carrying three mutant transgenes, i.e., amyloid precursor protein (Swedish, K670M/N671L), presenilin-1 (M146V), and tau (P301L), were used as an AD mouse model (Oddo et al., 2003). 3XTg-AD and C57BL/6J were acquired from the Jackson Laboratory (Bar Harbor, ME, United States). Mice were housed in our laboratory animal unit under 12-h light/dark cycles with food and ad libitum. YZT oral administration was started at 6 months of age up to 18 months of age 3XTg-AD mice fed with YZT diet admixture every day with a low dose (1 g/kg/d), a middle dose (2 g/kg/d), a high dose (4 g/kg/d), or vehicle. The body weight and in-cage behavior were monitored throughout the study.

### Rotarod Test

To evaluate motor function, mice were tested on the rotarod equipment (Harvard apparatus) as described previously (Monville et al., 2006). The rotarod test has been recognized widely and offers a simple evaluation of whole motor deficits in P301S tau mice and might provide a valuable quantitative test to assess the efficiency of therapeutic approaches (Rozas and Labandeira Garcia, 1997). In this study, we used a rotarod machine with system-controlled timer and sensors controlled in the software (Panlabs, Harvard Apparatus, MA, United States). Before the first training sessions, the mice were acclimated with a period of 3 min on the rotating drum. The fixed speed rotarod (FSRR) and accelerated rotarod (ARR) was described by (Dunham and Miya, 1957) to assess neurological deficits of motor function in mice. This process was reiterated every day for 3 min just prior to subsequent sessions for the training of the experimental animals. The rotation speed of the rod during the training period was increased every day with 4, 8, and 12 rpm during the test period. On the first day, the acceleration mode of rotarod was switched off, and the rotation was set in a fixed mode at a relatively low speed (4 rpm), to make the task easier for the animals as they learned. On the second and third day, the speed was increased to 8 and 12 rpm, respectively. Each mouse was subjected to three trials for everyday training and between each trial the trained mice were given 5 min gap with rest. In the final day, the trained mice were subject to evaluation in the rod that accelerated efficiently from 4 to 40 rpm over a period of 300 s. The latency of fall and the time taken for the latency of fall in the evaluation day was recorded automatically.

### Open Field Test

The procedure for the experiment was described previously (Zhang et al., 2014). A square box made of plexiglass (25 cm × 25 cm) was used as the open field apparatus to test the exploratory and locomotor function. The experimental animals were placed in the novel environment of the box and recorded the activities of the animal through the tracking camera. Locomotor functions such as time spent in central/marginal areas, rearing and fecal bolus were evaluated through an automated animal tracking system (Ethovision XT software Version 3.0, Noldus Information Technology, Leesburg, VA, United States). the (Noldus, Wageningen, The Netherlands).

### Morris Water Maze Test

MWM experiment was performed to evaluate the spatial memory, learning and recognition memory functions as described previously in our publications (Durairajan et al., 2017; Iyaswamy et al., 2020). The experimental animals were acclimatized in the behavior room, trained in visible platform, trained for six consecutive days in hidden platform, memory retention was evaluated in seventh day and monitored using the (Ethovision animal tracking software). All experimental animals in the study were included in the experimental training of visible platform for one day with four trials, continued with hidden platform training for 6 days, placing the platform in a constant location and the animals were placed in random spots of the tank for all trials for six consecutive days. In the seventh day the memory retention functions were evaluated in the probe trial using the animal tracking camera to analyze the time taken by the animal to probe the platform location and time spend in the platform quadrant by the experimental animal for the whole 60 min evaluation.

### Tau Extraction

The soluble and insoluble phospho tau from the brain homogenate of P301S tau and 3XTg-AD mice were prepared by us as per the procedure explained in our previous publications (Myeku et al., 2016; Iyaswamy et al., 2020). Here we have explained briefly about the different fractions of tau extraction in the following steps, the extracted brain was homogenized in 10 volumes of radioimmunoprecipitation assay (RIPA) buffer mixed with phosphatase inhibitors and protease inhibitors (Roche). The brain homogenate was centrifuged at 20,000 x g for 20 min to divide proteins into soluble fraction (S1) and the supernatant was incubated and rotated in tubes with 1% sarkosyl for 1 h at room temperature. Further to collect the insoluble fraction a high-speed ultracentrifugation (100,000 x g) for 60 min was employed to collect the insoluble proteins in the pellet fraction. The pellet was resuspended in 20 µL of Tris-EDTA, pH 8.0 labeled P2 (sarkosyl-insoluble tau) and supernatant was designated as S2 fraction (sarkosyl-soluble tau).

### Western Blot Analysis

The protein levels in the cell or brain homogenate was evaluated by the Western blot experiment as per the procedures explained in our previous publications (Durairajan et al., 2017; Iyaswamy et al., 2020). 5–10 µg of total protein in the brain or cell homogenate were separated on a SDS–PAGE gel as per the required percentage of gel depending on the target protein and blotted onto polyvinylidene difluoride (PVDF) membranes to detect the target protein namely PHF-1 (phospho-tau Ser396/Ser404), HT7 (total tau), AT8 (Phospho-tau Ser202/Thr205), CP13 (Phospho-tau Ser202), MC1 (a disease-specific conformational modification of tau), ALZ50 (misfolded tau) and *β*-actin (Table 1). The blot was blocked in milk (5%) and further probed with primary antibodies incubating overnight at 4°C in cold room with shaking. Next morning the blots were washed with tris-buffered saline, 0.1% Tween 20 (TBST) and incubated with target secondary antibodies for 2 h. The immunoblots were further enhanced with Supersignal -West Pico (Thermo Fisher Scientific, United States) and developed using X-ray film (Kodak).

**TABLE 1:** Specifications of antibodies used in the present study.

Antibody (clone)	Region specificity	Antigen	Source	Use and dilution
Rabbit polyclonal to APP CT695(CT695)	Human, mouse and rat FL-APP and CTFs	C-terminus 22 amino acid residues of *β*-APP peptide	Thermoscientific, Waltham, MA, United States	WB 1:1000
Biotinylated mouse monoclonal to human Aβ17–24 (4G8)	hAβ	Amino acids residues 17–24 of hAβ peptide	Biolegend, Dedham, MA, United States	IHC 1:500
Rabbit polyclonal to phosphorylated APP (Thr668)	Human phosphorylated APP at Thr668	Phospho epitopes matching to residues neighboring Thr668 of human APP695	Cell signaling, Danvers, MA, United States	WB 1:1000
AT100 monoclonal to phospho tau	Human, mouse and rat phospho tau	Epitopes matching to residues neighboring Thr212, Ser214 phosphorylated sites	Thermoscientific Waltham, MA, United States	IHC 1:500
AT8 monoclonal to phospho tau biotinated	Human, mouse and rat phospho tau	Epitopes matching to residues neighboring Ser 202, Thr 205 phosphorylated sites	Thermoscientific Waltham, MA, United States	IHC 1:500
HT7 monoclonal to total tau biotinated	Human specific	Human tau between residue 159 and 163	Thermoscientific Waltham, MA, United States	IHC 1:500
PHF-1 monoclonal to phospho tau	Human, mouse and rat phospho tau	Epitopes matching to residues neighboring Ser396 and Ser404phosphorylated sites	Prof. Peter Davies Albert Einstein College of Medicine, Manhasset, NY, United States	WB 1:1000
HT7 monoclonal to total tau	Human specific	Human tau between residue 159 and 163	Thermoscientific Waltham, MA, United States	WB 1:1000
Mouse monoclonal to *β*-actin (C4)	β-actin	Bird gizzard actin	Santa Cruz, Dallas,TX, United States	WB: 1:1000
ALZ50 monoclonal to phospho tau	Human, mouse and ratphospho tau	Epitopes matching to residues neighboring Phospho Ser phosphorylated sites	Prof. Peter Davies Albert Einstein College of Medicine,Manhasset, NY, United States	WB 1:1000
CP13 monoclonal to phospho tau	Human, mouse and ratphospho tau	Epitopes matching to residues neighboring Ser 202 phosphorylated sites	Prof. Peter Davies Albert Einstein College of Medicine,Manhasset, NY, United States	WB 1:1000
MC1 monoclonal to phospho tau	Human, mouse and rat phospho tau	Epitopes matching to residues neighboring Ser 312-322 phosphorylated sites	Prof. Peter Davies Albert Einstein College of Medicine,Manhasset, NY, United States	WB 1:1000
AT8 monoclonal to phospho tau	Human, mouse and rat phospho tau	Epitopes matching to residues neighboring Ser 202, Thr 205 phosphorylated sites	Thermoscientific Waltham, MA, United States	WB 1:1000
AT180 monoclonal to phospho tau	Human, mouse and rat phospho tau	Epitopes matching to residues neighboring Thr 231 phosphorylated sites	Thermoscientific Waltham, MA, United States	WB 1:1000

### Immunohistochemical Analysis

Immunohistochemical analysis was performed as per the procedure described by us in the previous publications (Durairajan et al., 2017; Iyaswamy et al., 2020). The experimental mice were anesthetized and perfused with 1X Phosphate buffered saline (PBS) and then brain was dissected. The extracted brains were fixed with 4% paraformaldehyde at 4°C for 2 days and then cleaned with 1XPBS for two times further soaked in 30% sucrose at 4°C till we finally embedded in optimal cutting temperature (OCT) medium. The cortico-hippocampal region were sectioned using Shandon Cryotome SME Cryostat (Ramsey, MN, United States) at 30 μm intervals and stored at 4°C with PBS, 0.1% Tween 20 (PBST); further blocked for 2 h at room temperature with 5% normal bovine serum albumin (BSA) in 1 X PBS and then incubated with target primary antibodies namely MC1, AT100 (Phospho-tau Thr212/Ser214) and AT8 for P301S mice and AT8, HT-7, PHF1 and 4G8 (Aβ17-24) for 3XTg mice (1:100 dilute in 2% BSA) overnight at 4°C. Next day the brain slices were washed in 1XPBS and then for fluorescent staining the brain slices were incubated with secondary fluorescent antibody green or red probes (Dilute as 1:500 blocking buffer) for 2 h. The floating brain slices were fixed in the coated slides, air-dried out and mounted with fluorescence mounting medium (Fluorsave, Sigma-Aldrich).

### Cell Culture

SH-SY5Y cell line expressing tau P301L (SH-SY5Y-P301L) was generated using lentiviral gene transfer; this cell line was donated by Dr. Lars Itner (University of Sydney, Australia) (Ke et al., 2012). The SH-SY5Y-P301L cells cultured in DMEM-F12 (Thermo sceintific) containing 15% of heat inactivated fetal bovine serum (FBS) and 1X PSN (Invitrogen) with 3–5 μg/ml of blasticidin (IThermo sceintific), a nucleoside antibiotic used as a selection marker of tau P301L. Chinese hamster ovary (CHO) cell line expressing human APP751 with V717F mutation (7PA2) cells was gifted by Dr. Edward Koo, University of California, San Diego. 7PA2 cells secrete Aβ oligomers rapidly after Aβ peptide generation inside particular intracellular vesicles are successively secreted to the medium (Podlisny et al., 1995).

### RNA Extraction, Microarray Processing, and Data Analysis

The SH-SY5Y-P301L cells were seeded 1.2 × 10^6^ cells/dish and after incubating for 16–20 h, the SH-SY5Y-P301L cells were then treated by 0.1% dimethyl sulfoxide (DMSO) (vehicle control group) and YZT (100 μg/ml), respectively, for 6 h. Cells were harvested using Trizol (TRI Reagent Solution, Ambion) and then stored at -80°C overnight, followed by Phalanx array processing (Phalanx Biotech Group’s CytoOneArray) in the next day. The differentially expressed genes with 2-fold changes as compared to vehicle control were used to query Clue (https://clue.io/) (Subramanian et al., 2017), which is a gene expression profile-based bioinformatics database. These differentially expressed genes were used as query inputs to reveal the hubs in the network using Ingenuity Pathways Analysis (IPA) and search tool for interactions of chemicals (STITCH (https://www.qiagenbioinformatics.com/products/ingenuity-pathway-analysis/ and chemical- protein interaction networks, http://stitch.embl.de).

The Connectivity Map (CMap) project (Lamb et al., 2006) was established to accommodate a huge number of gene-expression profiles of various studies of bioactive small molecules to give design-matching algorithms to find these data. Briefly, a potent optimistic connectivity score (similarities) suggests that the profile in CMap displays the gene expression like the query. Many herbal medicines have performed microarray profiling and then executed the bioinformatics analysis via CMap to explore the mechanism of actions (Toyoshiba et al., 2009). By using Clue, which is a new CMap database (https://clue.io/) (Subramanian et al., 2017), we could obtain a list of small molecules, shRNA and overexpression constructs sharing similar gene expression patterns with YZT-treated cells for us to predict the mechanism of actions of YZT.

### Statistical Analysis

All the raw data was processed as per the requirements of group comparison and number of animals per group are presented as mean ± SEM or SD. Most of the experiments of biochemical assays like immunoblot and immunohistochemistry were evaluated by “one-way ANOVA” assessment. However, Behavior study was evaluated by “2-way analysis of variance (ANOVA)” because at the same time between the groups and different timepoint were compared. “Pair-wise differences between groups were compared using either Bonferroni’s or Fisher’s least significant difference (FLSD)-post hoc multiple comparisons test”. Graphical demonstration and statistics were performed with “GraphPad Prism 6 (GraphPad Software, San Diego, CA, United States)”.

## Results

### Quality Analysis of Yuan-Hu Zhi Tong, *Corydalis yanhusuo* and *Angelica dahurica* by LC- QTOF/MS

**FIGURE 1:**
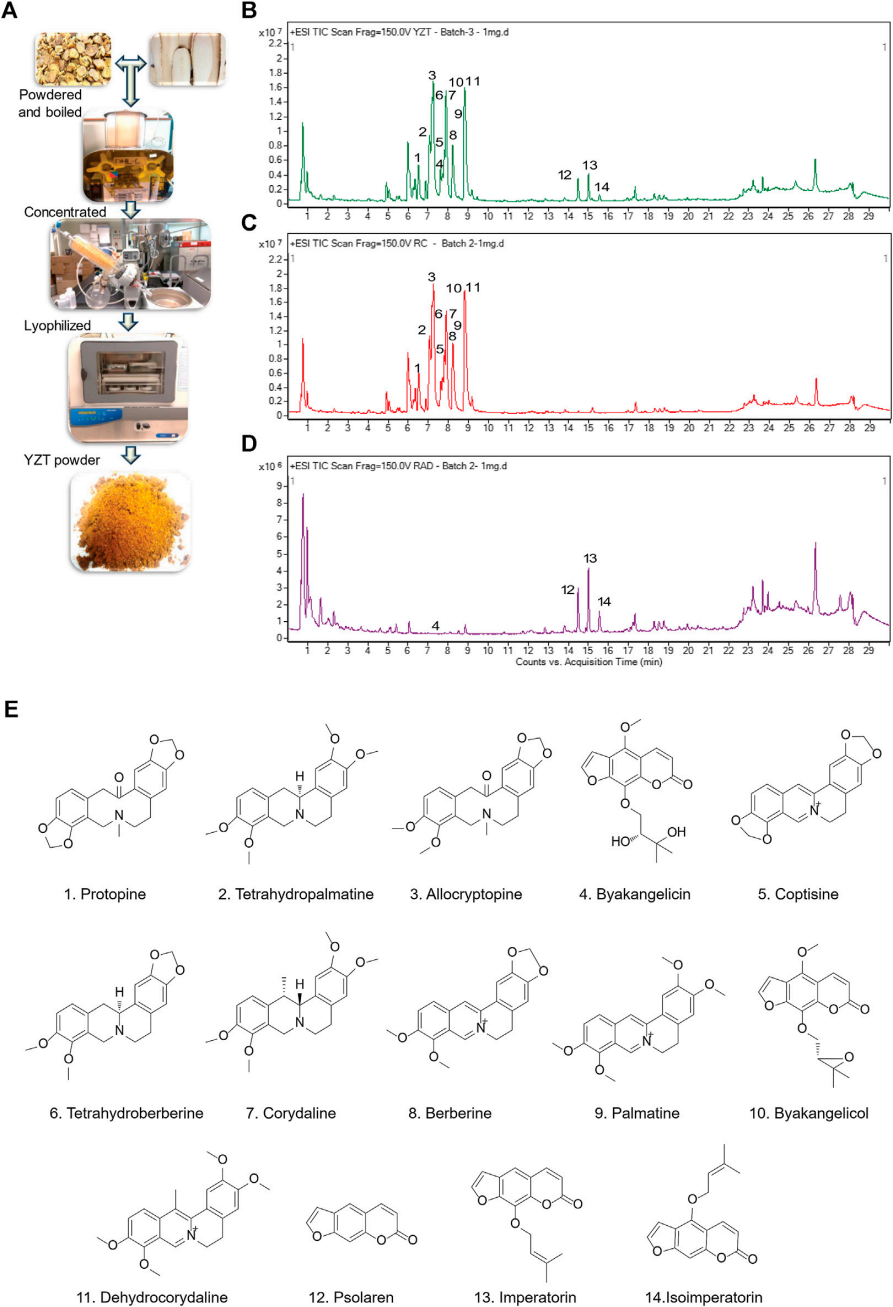
Qualitative analysis and herbal preparation of YZT. **(A)** The herbal materials of YZT were powdered boiled in water; the extract was concentrated and lyophilized to prepare YZT extract powder. LC-ESI-Q/TOF chromatograms (TIC) of **(B)** YZT, **(C)** CY, and **(D)** ADH. **(E)** Peaks: 1. Protopine, 2. Tetrahydropalmatine, 3. *α*-Allocryptopine, 4. Byakangelicin, 5. Coptisine, 6. Tetrahydroberberine, 7. Corydaline, 8. Berberine, 9. Palmatine, 10. Coptisine, 11. Dehydrocorydaline, 12. Psolaren, 13. Imperatorin, and 14. Isoimperatorin.

We first prepared YZT by mixing CY and ADH in the ratio of 2:1. Dried raw material of the herbs were pulverized into powder, steeped in 80% alcohol overnight, filtered, concentrated by rotary evaporation, and then finally subjected to lyophilized powder (Figure 1A) and analyzed YZT, CY and ADH by LC-QTOF/MS. The chromatograms of YZT, CY and ADH are shown in Figures 1B–D. The highly abundant 14 compounds among the 24 peaks were detected and verified (Figure 1E) by comparing to the internal standards with high-resolution MS and MS/MS fragmentation was presented.

### Yuan-Hu Zhi Tong Treatment Reverses Motor Impairment in P301S Tau Mice and Ameliorates Learning and Memory Functions in 3XTg-AD Mice

**FIGURE 2:**
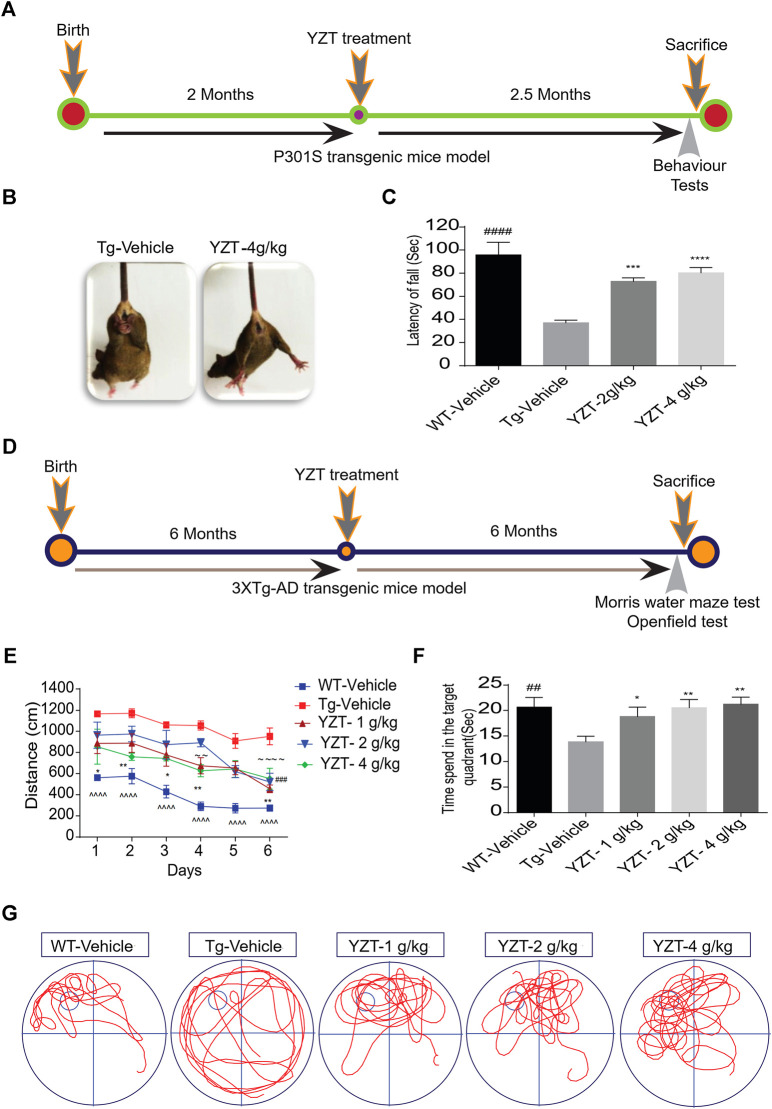
YZT treatment improved motor function and enhances learning and memory function in P301S and 3XTg-AD mice models. **(A)** The timeline schedule of drug administration and behavior tests in P301S tau mice. **(B)** In tail hanging test, YZT improved the motor function of 4 months P301S tau mice when compared to vehicle. **(C)** In rotarod test, regular treatment of 2-month old P301S tau mice with YZT (2 or 4 g/kg) via food admixture for 2 months significantly ameliorated motor function. The average latency of mice (N = 14) fall was calculated. ####*p* < 0.01 (WT/Vehicle), ***p < 0.001(YZT 2 g/kg/d), and ****p < 0.0001 (YZT 4 g/kg/d) vs Tg-vehicle. **(D)** The timeline schedule of drug administration and behavior tests in 3XTg-AD mice. **(E)** After the visible platform training in Morris water maze test, the experimental mice were trained for 6 days with four trials per day to learn the place and location of hidden platform in the tank. The learning potential of the YZT treatment group improved with the time and days of learning when compared to the Tg-vehicle. Each point represents the mean length values of four trials per day, (N = 14). ^^^^*p* < 0.001 (WT-vehicle vs. Tg-vehicle); ###*p* < 0.001 (YZT -1 g/kg vs. Tg-vehicle), ∼∼∼∼ *p* < 0.0001 (YZT-2 g/kg vs. Tg-vehicle), **p < 0.01 (YZT-4 g/kg vs. Tg-vehicle). **(F)** on the seventh Day, the probe trial demonstrated that YZT treatment showed improved memory function in probing the platform in target quadrant when compared to the Tg-vehicle. **(G)** The displayed pictures illustrate YZT improved the memory retention in animal’s behavior during the probe trial in the animal tracking camera videos.

We utilized 2-month old P301S tau (N = 14) mice, i.e before the onset of tau pathology, to evaluate the efficacy of YZT. The timeline indicates the period of drug administration and behavior tests plan in P301S tau mice model (Figure 2A). We found that chronic drug-feed administration of YZT (2 or 4 g/kg/d) for nearly 2.5 months until 4.5 months of age, YZT did not substantially alter animal body weight or affect any prominent harmful impacts in P301S tau mice model (Supplementary Figure S1A). YZT treatment significantly ameliorated motor impairment compared with control mice, as inferred from the tail hanging test (Figure 2B). Further we employed the rotarod experiment to assess the motor function and locomotor activities of vehicle group and YZT treatment group. Substantial changes were detected among the YZT (2 and 4 g/kg/d)-treated mice and vehicle group in the latency to fall (Figure 2C). These findings suggest that YZT improved motor function and enhanced locomotor activity.

We tested the long-term effect of YZT in another AD mice model, 3XTg-AD, via food admixture. The timeline for drug administration and behavior tests plan in 3XTg-AD mice is displayed in Figure 2D. We found that YZT treatment did not affect the body weight of 3XTg-AD mice (Supplementary Figure S1B) during the whole study. Furthermore, in behavioral studies, we found that YZT administration for 6 months significantly reduced the distance traveled by 3XTg-AD mice to detect the platform during the training in the “Morris Water Maze test (MWM)” (Figure 2E), The transgenic 3XTg-AD vehicle group (Tg-vehicle) took a lengthier route to find the platform compared to vehicle-treated wild-type (WT) mice (Figure 2E). However, YZT treatment in 3XTg-AD mice clearly improved the learning ability, as evidenced by the shorter path on the fourth and sixth days of the learning test (Figure 2E). Tg-vehicle mice consistently traveled a longer path [F (5,15) = 13.22; *p* < 0.0001] when compared with the vehicle-treated WT mice during all six training sessions (post-hoc, *p* < 0.0001).

To assess memory function after the 6-days learning, we did a probe trial 24 h after the sixth hidden day. During the probe trial, the YZT-treated group spent more time in probing the platform compared to the Tg-vehicle group in the target quadrant (Figure 2F). “One-way ANOVA analysis” of the probe test, the search ratio in the target quadrant indicated a significant effect of YZT treatment of 3XTg-AD mice on memory retention compared to the Tg-vehicle [F (4, 64) = 3.637; *p* < 0.01]. Altogether, these findings reveal that spatial learning and memory functions of 3XTg-AD mice is improved by YZT administration.

The open field experiment was conducted to assess the exploratory and locomotor activity of Tg-vehicle group and YZT-treated group. There were no significant variations between the Tg-vehicle and YZT-treated group in total moving distance and total ambulatory movement duration, although YZT-treated groups performed slightly better than the Tg-vehicle group (Supplementary Figures S1C,D).

### Yuan-Hu Zhi Tong Administration Relieves Tau Pathology and Lowers Phospho Tau Load in P301S Tau and 3XTg-AD Mice Brain

**FIGURE 3:**
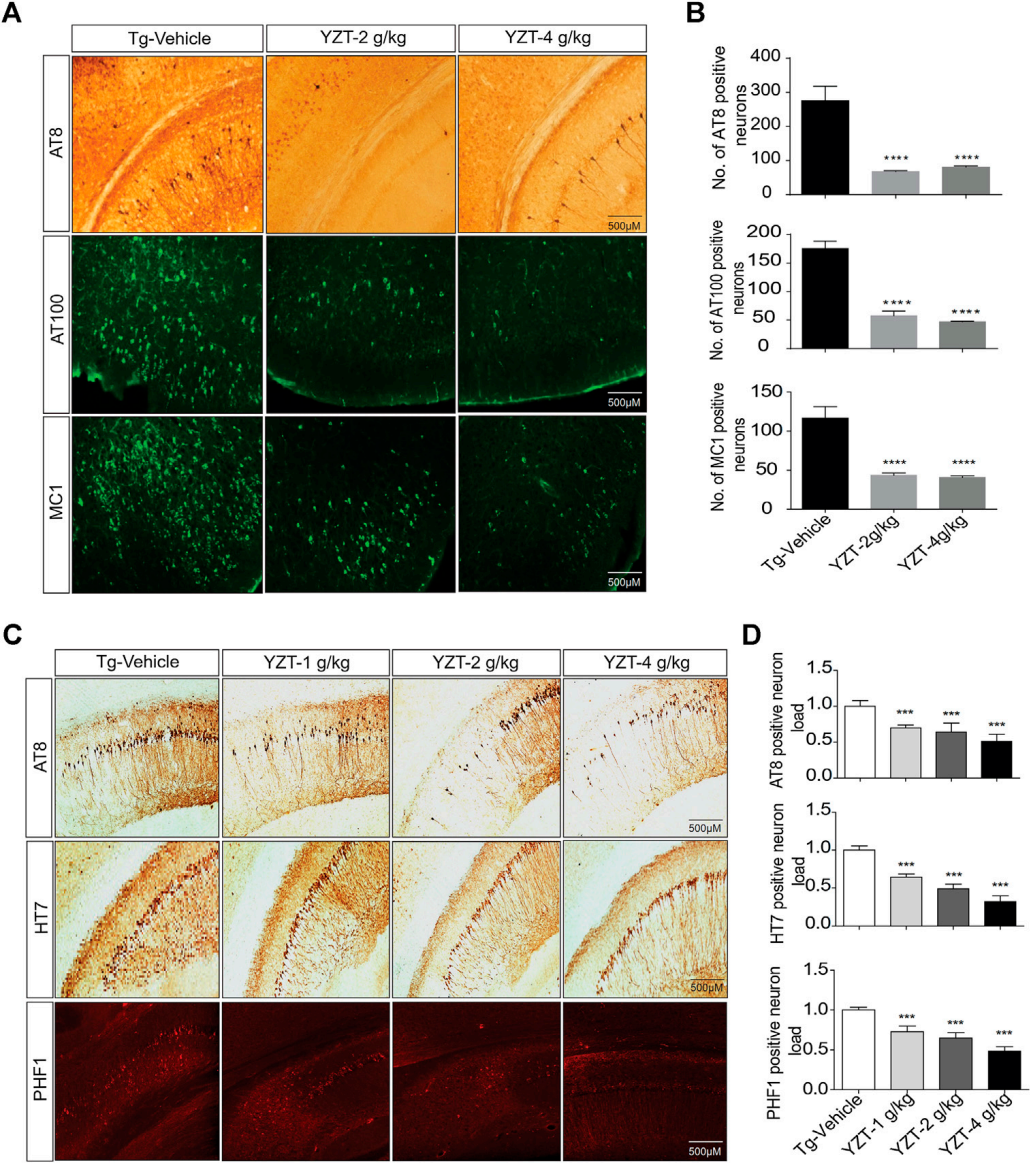
YZT treatment mitigates tau pathology in P301S and 3XTg-AD mice. **(A)** Chronic treatment of YZT reduced AT8, AT100 and MC1 in the cortico-hippocampal brain region of P301S tau mice. The displayed pictures are fluorescent images of brain slice taken in fluorescent detecting microscope. **(B)** The quantified results of AT8, AT100 and MC1 positive neurons in the cortico-hippocampal brain region of P301S tau mice was performed using Image J software N = 14, **p < 0.01; ***p < 0.001. **(C)** Chronic treatment of YZT reduced AT8-, PHF- and HT7-positive neurons in the CA2, CA3 hippocampal region of 3XTg-AD mice. **(D)** Quantification of AT8-, HT7- and PHF1 positive neurons in the CA2, CA3 hippocampal brain region of 3XTg-AD mice. The number of phospho and total tau-positive neurons were quantified using ImageJ software N = 4.

To evaluate the tau pathogenesis in the P301S tau mice model, AT8, AT100 and MC1 monoclonal antibodies were used. The AT8 epitope indicates the phosphorylation site of Thr212/Ser214 signifies internal repeats of “microtubule binding domains (RT-14)”. The epitope AT100 is also located outside the RT-14 and. The epitope MC1 is located in tau 5-15/312-322 all these epitopes imply the development of tau pathogenesis. AT8-positive immunostaining in the cortico-hippocampal brain region of P301S tau mice demonstrated that YZT treatment at doses of 2 and 4 g/kg/d decreased AT8 positive cell count in cortico-hippocampal region by 74% (*p* < 0.05) and 70%, respectively (Figures 3A,B). Immunostaining of AT100-positive neurons also revealed that YZT treatment at doses of 2 and 4 g/kg/d decreased AT100 positive cell count in cortico-hippocampal region by 68 and 74%, respectively (Figures 3A,B). Immunostaining of MC1-positive neurons also revealed that YZT treatment at doses of 2 and 4 g/kg/d decreased MC1 positive cell count in cortico-hippocampal region by 68% (*p* < 0.05) respectively (Figures 3A,B).

3XTg-AD mice brain slice was immune stained with AT8, PHF-1 and HT7 monoclonal antibodies. The epitope PHF-1 is also located outside the RT-14 and requires the phosphorylation of paired helical filaments pSer396/pSer404 and aberrant conformation of tau. The PHF1-positive neuron load in the brain slice of 3XTg-AD mice demonstrated that YZT treatment at doses of 1, 2, and 4 g/kg/d decreased PHF1 positive cell count dose dependent in CA2, CA3 hippocampal region by 44, 55, and 70%, respectively (*p* < 0.05) (Figures 3C,D). The AT8, HT7-positive neuron load in the brain slice of 3XTg-AD mice indicated that YZT treatment at doses of 1, 2, and 4 g/kg/d decreased AT8, HT7 positive cell count dose dependent in CA2, CA3 hippocampal region by 48, 58, and 74%, respectively (*p* < 0.05) (Figures 3C,D). Long-term YZT treatment did not significantly reduce 4G8 positive neurons in 3XTg-AD mice CA2, CA3 hippocampal brain region are shown in Supplementary Figures S2C,D. The quantification of plaque load was performed using ImageJ software.

### Yuan-Hu Zhi Tong Decreases the Insoluble Tau Load in the Brain of P301S Tau and 3XTg-AD Mice

The above findings of decreased phospho-tau positive neurons were further corroborated by the differential separation of insoluble tau. The insoluble tau extraction in the brain homogenate of P301S Tau mice model is described previously (Myeku et al., 2016). The brain sample was first extracted with RIPA buffer. The resulting RIPA fraction was further extracted with 1% sarkosyl detergent and ultra-centrifuged (100,000 x g) at least 1 h. Resulting supernatant was marked as soluble tau and pellet was designated as insoluble tau fraction.

**FIGURE 4:**
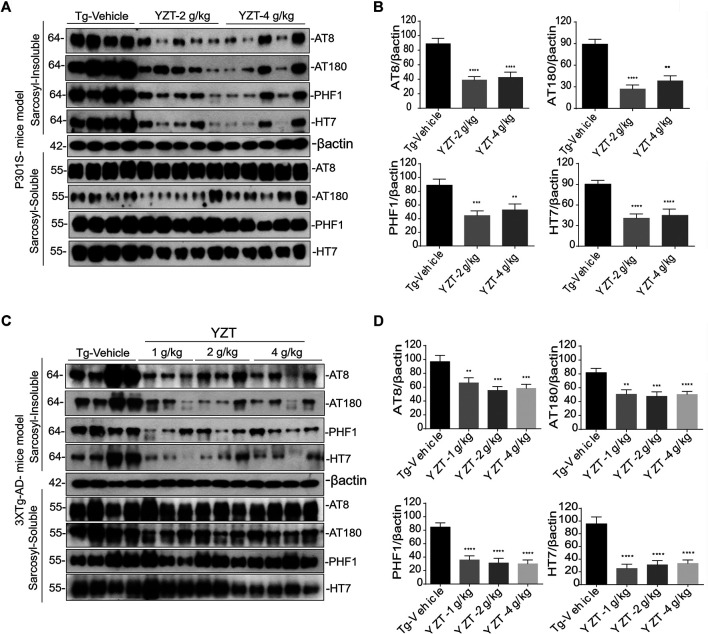
YZT reduces the pathological phospho tau in P301S and 3XTg-AD mice models. **(A)** Regular YZT treatment decreased the sarcosyl insoluble tau protein levels in the brain homogenates of P301S tau mice. There was no change in the soluble tau in the sarcosyl soluble brain homogenate fraction. **(B)** The quantified results of different tau epitopes protein levels in brain lysates of P301S tau mice was performed using Image J software. **(C)** Long term YZT treatment lowered the sarcosyl insoluble phospho tau protein levels in the brain homogenates of 3XTg-AD mice model. There was no change in the soluble tau in the sarcosyl soluble brain homogenate fraction. **(D)** The densitometric analysis of the various tau epitopes protein levels in the brain lysates of 3XTg-AD mice models was performed using Image J software. **p < 0.01; ***p < 0.001.

AT8, AT180, CP13, PHF-1 antibodies were used to detect the phosphorylated tau in both the fractions of soluble and insoluble toxic protein aggregates. Misfolded conformation tau and total tau were detected using Alz50 and HT7 antibodies. There were no significant differences in phosphorylated, misfolded and total tau in the soluble fraction of the three groups (Figure 4A). In contrast, phosphorylated, misfolded and total tau were significantly reduced in the sarkosyl-insoluble fraction (Figures 4A,B) of YZT-treated groups compared to that in the vehicle-treated group.

To further confirm, we investigated 6 months treatment of YZT in 3XTg-AD mice, treated mice were sacrificed and levels of soluble and insoluble tau in homogenates of their whole brain hemispheres were determined. YZT-treated groups showed significant reduction in levels of insoluble phospho tau when compared with vehicle-treated animals (Figures 4C,D). Notably, there were no significant differences in phosphorylated, misfolded and total tau in the soluble fraction of the three treatment groups (Figure 4C). In contrast, phosphorylated, misfolded and total tau was significantly reduced in the sarkosyl-insoluble fraction (Figures 4C,D) of YZT-treated groups but not in the vehicle-treated group of 3XTg-AD mice. Long-term YZT treatment did not significantly reduce APP, CTFs and its phosphorylated form in the SDS brain fraction of 3XTg-AD mice. Representative figures of APP, CTFs and its phosphorylated form in the SDS brain fraction of 3XTg-AD mice are shown in Supplementary Figures S2A,B.

### Microarray and Connectivity Maps

**FIGURE 5:**
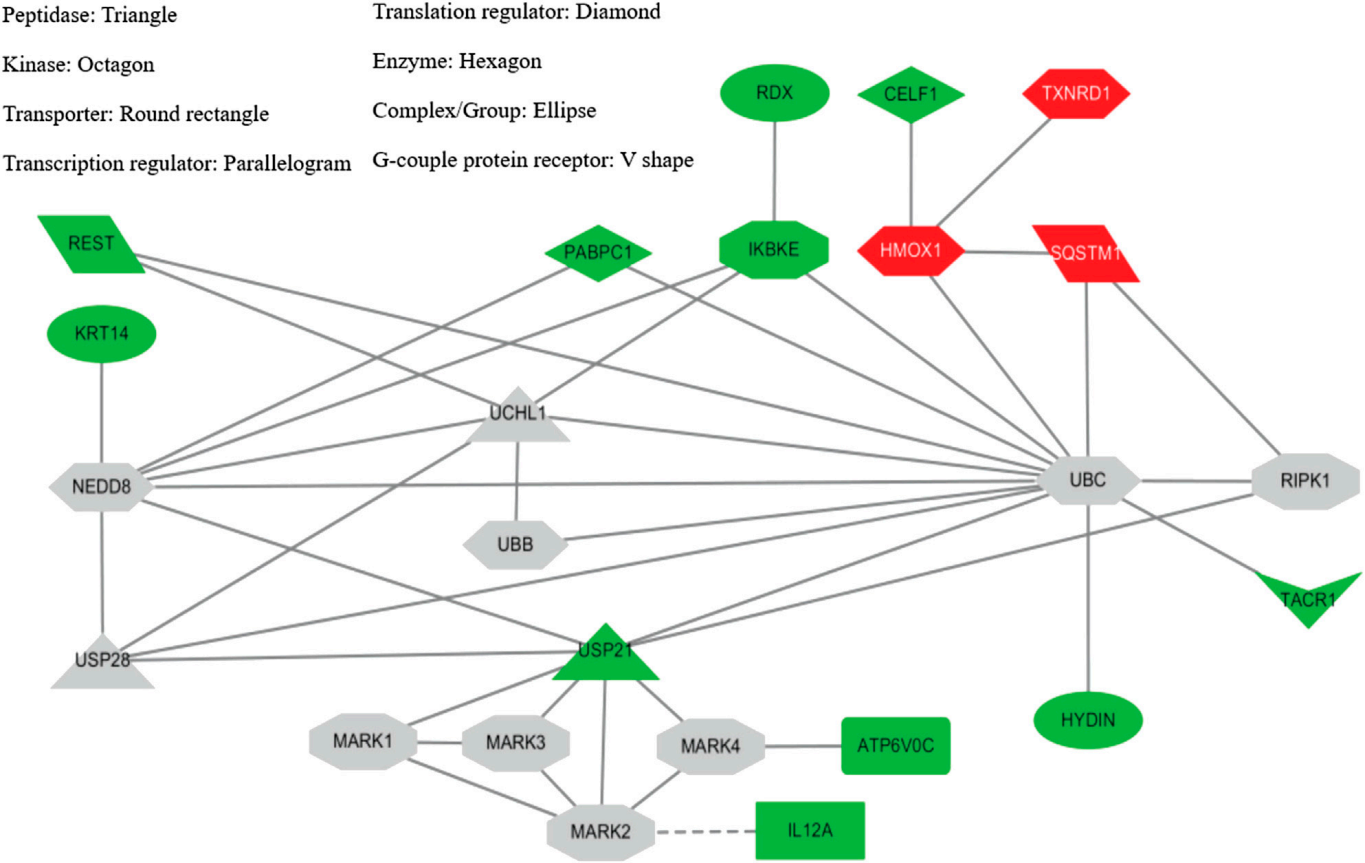
YZT-mediated network analysis reveals UPS as potential target. **(A)** Potential network involved in YZT-treated SHSY5Y-P301L cells. The gene expression profile of YZT-treated SHSY5Y-P301L cells were analyzed by using Ingenuity Pathway Analysis (IPA and STITCH (http://stitch.embl.de/). Red: up-regulation. Green: down-regulation.

In SH-SY5Y-P301L cells, when compared to vehicle control, the YZT treatment of 100 μg/ml in cells demonstrated a 2-fold upregulation of 13 genes with down-regulation 64 genes graphed in a heat map (Supplementary Figure S3). The up- and down-regulated genes were used to query Consensus Path Database (CPDB) (CPDB is an integrative interaction database that gathers molecular interaction data integrated from 32 different public repositories and provides a set of computational methods and visualization tools to explore these data), IPA (Ingenuity Pathway Analysis, QIAGEN), STITCH (search tool for interactions of chemicals), and Clue (This platform provides integrated access to datasets, results from the processing and analysis of these data, and software tools that the community can leverage to advance their research), respectively. From IPA analysis, treatment of YZT generated an upregulated network center on HMOX1 (heme oxygenase 1, an essential enzyme in heme catabolism, cleaves heme to form biliverdin), SQSTM1 (sequestosome 1 or p62) and TXNRD1 (thioredoxin reductase 1) (Figure 5, upper right label in red). Analysis from CPDB suggests that these three hub genes, HMOX1, SQSTM1, and TXNRD1, are involved in the Nuclear Receptors Meta-pathway and Nuclear factor (erythroid-derived 2)-like 2 (NRF2)-mediated oxidative stress response.

Using the gene expression signature from YZT treatment as an input query, Clue analysis gave a connectivity score (or similarity) of 96.51 to upregulation of SQSTM1 and 96.17 to NF-kB pathway inhibitors (Figure 5). In fact, treatment with YZT resulted in the upregulation of SQSTM1, which can bind to ubiquitin (Lee and Weihl, 2017). Using STITCH dataset, an extended protein-protein interaction network (label in gray) was generated (Figure 5) and ubiquitination was highlighted from these 24 genes.

### Yuan-Hu Zhi Tong Reduces Phospho- Tau via Ubiquitin Proteasomal System *In Vitro* and *In Vivo*

**FIGURE 6:**
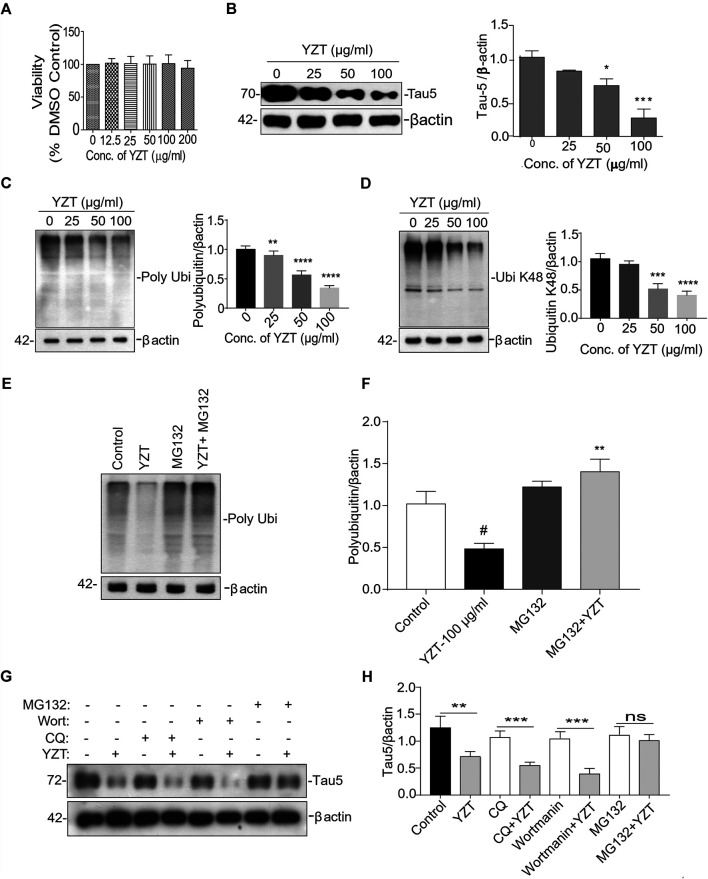
Involvement of UPS in YZT-mediated degradation of tau. **(A)** Viability test of YZT on SH-SY5Y-P301L cells. Cell viability was determined using the MTT assay. **(B)** Effect of YZT on the level of total phospho tau (tau 5) in SH-SY5Y-P301L cells. **(C)** YZT significantly decreased the levels of polyubiquitin in SH-SY5Y-P301L treated cells. **(D)** YZT significantly decreased the levels of ubiquitin K48 in SH-SY5Y-P301L treated cells. **(E,F)** YZT reduces the level of insoluble phosphorylated and misfolded tau via UPS mediated degradation pathway. YZT significantly reduced the levels of polyubiquitin in SH-SY5Y-P301L treated cells. The reduction of polyubiquitin was restored when the cells were pretreated with MG132 (proteasome inhibitor), thus clearly illustrating that involvement of UPS in YZT-mediated degradation of tau. **(G,H)** YZT- treated SH-SY5Y-P301L cells in the presence of autophagy inhibitors (CQ and wortmannin) the tau reducing effect of YZT was not blocked but in the presence of MG132 blocked the YZT’s tau reducing effect. All the results were represented as mean ± SEM of three independent experiments. N = 3, **p < 0.01; ***p < 0.001.

Further to evaluate whether tau-reducing activity of YZT is via ubiquitin proteasomal system (UPS), we carried out experiments *in vitro*. First to confirm the cytotoxicity of the ethanolic extract of YZT, different concentrations of extracts were added to SH-SY5Y P301L and 7PA2 cells for 48 h. The YZT did not show any adverse effect in the cell morphology and cell viability (Figure 6A) as demonstrated by MTT assay in SH-SY5Y-P301L and 7PA2 cells (Supplementary Figure S4A).

We further evaluated its tau-reducing activity in SH-SY5Y-P301L cells following YZT treatment with three different doses (25, 50, and 100 μg/ml final concentration) for 48 h. As expected, the treatment with YZT significantly and dose-dependently reduced the levels of total tau in SH-SY5Y tau-mutant P301L cells (Figure 6B) without influencing the viability of cells. The low concentration of YZT (25 μg/ml) insignificantly reduced the abnormal tau level by 12% in SH-SY5Y P301L cell lysates (Figure 6B). At 50 and 100 μg/ml, abnormal tau was reduced by 33% and by 79%, respectively, compared to the vehicle (0.1% DMSO) control (Figure 6B). These data indicate that YZT indeed reduces abnormal tau aggregation in the cell model of tau, and it does so at a concentration-dependent manner YZT treatment did not significantly reduce the level of APP, C-terminal fragments (CTFs), soluble APP (sAPP)α and sAPPβ in the cell lysate of 7PA2 cells (25, 50, and 100 μg/ml final concentration), for 48 h (Supplementary Figure S4B).

**FIGURE 7:**
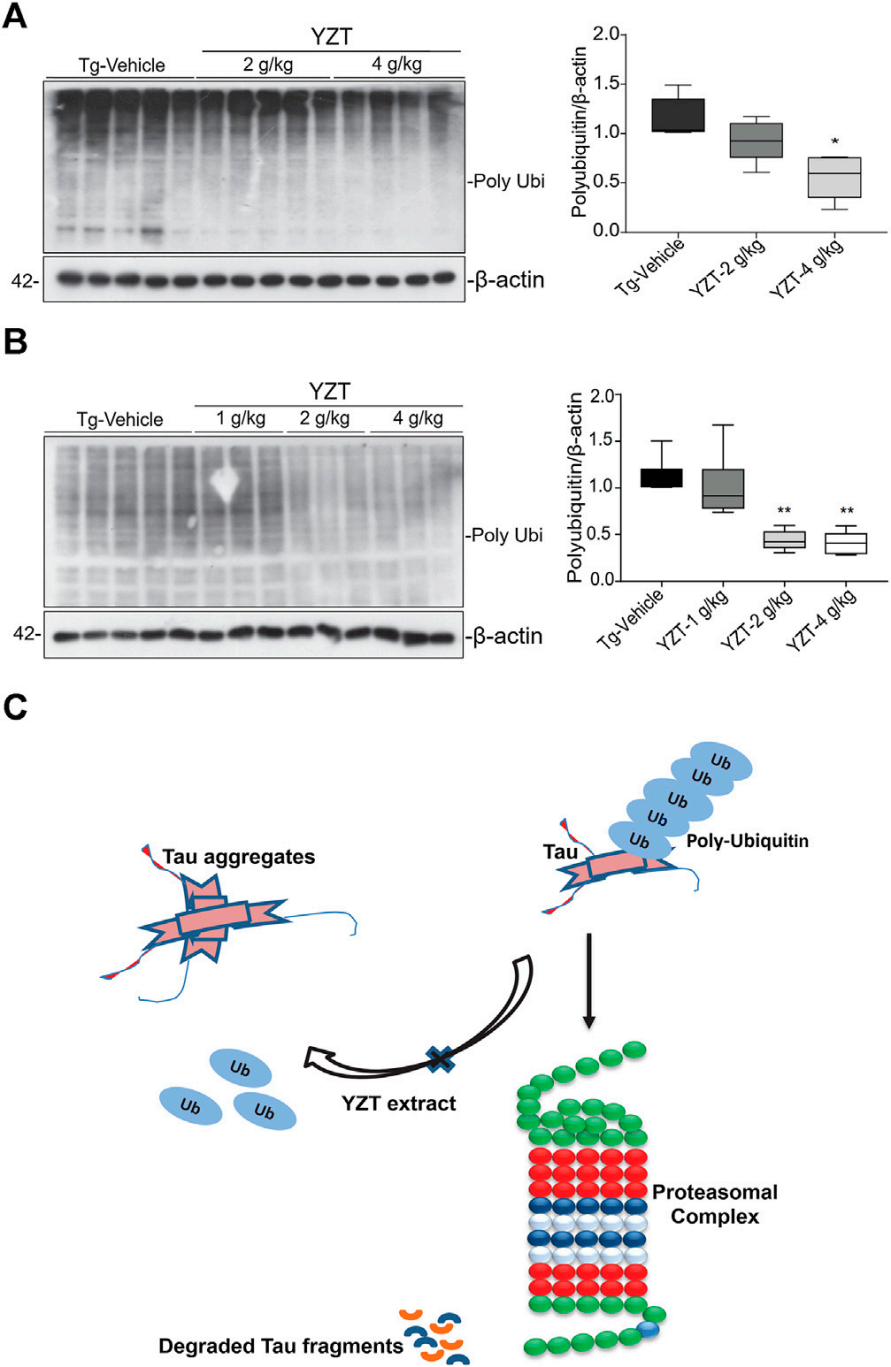
Schematic representation of YZT-mediated UPS pathway *in vivo*. **(A)** YZT treatment significantly decreases the poly ubiquitin levels in P301S mice brain homogenate compared to the Tg-vehicle and its quantification. **(B)** Long term YZT treatment in 3XTg-AD mice significantly reduced the ploy ubiquitin protein levels when compared to Tg-vehicle. **(C)** Schematic diagram illustrating that involvement of ubiquitin proteasomal pathway in YZT-mediated degradation of tau.

It is well known that the enzyme-determining step in the ubiquitin proteasome system is the ubiquitination of protein substrates and followed by digestion of all ubiquitinated proteins that bind to proteasome. We attempted to investigate whether YZT modulated the total and the K-48 specific ubiquitinylated conjugates in SHSY5Y-P301L-tau cells. The amount of both total (Figure 6C) and K-48 ubiquitinated proteins was dose-dependently lowered by the YZT treatment (Figure 6D), which is consistent with the microarray data. Because MG132 (proteasome inhibitor) stopped the effect of YZT on polyubiquitin degradation (Figures 6E,F) and YZT did not influence autophagy as evidenced by the level of light chain 3 (LC3)-II in a time dependent and dose dependent effect (Supplementary Figures S4C,D). Further to evaluate that YZT effect on tau degradation involving UPS and independent of autophagy, we used the inhibitors of Autophagy (chloroquine (CQ) and wortmanin) and UPS (MG132) with YZT treatment to identify the tau degradation pathway. We did not find any increase in the LC3-II levels in the presence of CQ and wortmanin with YZT treatment, which clearly illustrates tau degradation is independent of autophagy (Supplementary Figure S4E). In addition, in the presence of autophagy inhibitors (CQ and wortmanin) the YZT’s tau reducing effect was not blocked but MG132 blocked the YZT’s tau reducing effect (Figures 6G,H). Which clearly indicate that proteasomal degradation was exclusively responsible for YZT’s effects on ubiquitylated protein degradation of tau. Further illustrating the involvement of UPS in YZT-mediated degradation of tau. Further to correlate this finding with the *in vivo* study, we did the western blot experiment to elucidate the protein expression of UPS proteins in transgenic animal model brain homogenate. Surprisingly, the YZT-treated P301S (Figure 7A) and 3XTg-AD (Figure 7B) brain lysates illustrated a significant decrease in polyubiquitin proteins compared to the Tg-vehicle group, demonstrating YZT degrades the insoluble phospho tau via UPS both *in vitro* and *in vivo.* Further we also evaluated the other degradative pathway proteins in YZT-treated mice brain lysates compared to the Tg-vehicle group, we did not discover any substantial alterations in autophagy proteins and kinases (Supplementary Figures S5A,B) involved in tau degradation. The schematic representation depicts the involvement of UPS in YZT-mediated degradation of tau (Figure 7C).

## Discussion

YZT administration to the *in vivo* tau mice model demonstrated a strong tau reducing effect clearly illustrating the therapeutic efficiency of the Chinese herbal medicine. Until now, the actual medicinal value of YZT in curing AD pathology and cognitive deficits is not validated. The present study demonstrates that YZT administration in P301S and 3XTg-AD mice models reduced the tau deposition and improved both motor co-ordination and memory retention, respectively. The herbal composition of YZT is a combination of CY and ADH in a perfect ratio 2:1 and at this specified ratio YZT significantly decreased insoluble phospho tau including misfolded and total tau in brain fractions. Chronic YZT administration boosted the motor function as demonstrated by rotarod experiments and recovered the P301S tau mice hindlimb paralysis compared to the Tg-vehicle. Notably, YZT oral administration improved the motor and memory functions in addition to a substantial reduction in the insoluble tau burden.

Successive extraction of brain homogenates by RIPA and sarkosyl detergent is a method commonly used with different AD mouse models to assess the - total amount of homogenous tau and insoluble tau species in brains (Durairajan et al., 2012; Dillon et al., 2013). During this study, we observed that different doses of YZT significantly reduced sarkosyl-insoluble tau in the brains of P301S tau and 3XTg-AD mice. Overall, the YZT-induced reduction of insoluble tau (65–75%) in the brain hemispheres corresponds well with the decrease in the AT8-positive load (33%) reflecting the overall reduction of tau load in the whole brain. “Since a progressive shift of brain tau from soluble to insoluble pools plays a mechanistic role in the onset and/or progression of AD (Geerts et al., 2013)”, YZT mediated decrease in insoluble phospho tau clearly demonstrates its ability to delay the onset of tau pathogenesis in AD.

The TCM herbal formula, YZT is clinically used to treat pain and neuralgia (Yuan et al., 2004; Xu et al., 2013). Neuropharmacological studies of YZT and its components have only confirmed the anti-acetylcholine esterase and anti-depressive activities (Xu et al., 2013); for the first time we have found that YZT'|’s components show anti-tau activities. Although “AD is commonly considered a memory disorder, almost all people diagnosed with AD develop neuropsychiatric symptoms” (NPS: anxiety, motor impairment, hallucinations, and psychosis) at some stage during their disease (Podlisny et al., 1995). Recent Aβ-based therapies do not prevent cognitive decline, NFT formation or neurodegeneration (Dillon et al., 2013). Recent studies have also highlighted the strong correlation between tau pathology, cognitive decline and NPS (Yoshiyama et al., 2013).

Conventional AD drug discovery has taken a great approach in developing many clinical trials to halt the pathogenesis as an established target. Drug discovery for AD based on the cholinergic and *β*-amyloid hypothesis has not succeeded in that medications anticipated to treat acetylcholine deficiency and *β*-amyloid accumulation have not been effective (Yoshiyama et al., 2013). Based on these failures, and because NFTs are well correlated with cognitive impairment and NPS (anxiety, motor impairment, hallucinations, and psychosis) (Geerts et al., 2013), current disease-modifying approaches are taking a new approach, focusing on tau-based pathologies. In AD patients many microarray and genetic studies on tau-associated pathogenesis has elucidated the link of psychiatric disorders (markers of psychosis and mood disorders) (Altar et al., 2009).

Traditional Chinese medicine uses a broad way of pharmacological approach in curing neurodegenerative diseases by combining a few herbs with multifactorial disease modifying efficacy. Therefore, TCM typically uses this type of combinational, holistic approach in curing disease and YZT is the appropriate TCM candidate to tackle multifactorial symptoms of AD including tau aggregation. The disease-modifying activity of YZT against AD and other neurodegenerative disease in general or tauopathies have never been studied. We believe that YZT has the potential to be an effective therapeutic TCM that will both ameliorate the cognitive and psychiatric symptoms and address the underlying biochemical causes of AD.

In an experimental plan primarily designed to identify anti-Aβ and anti-tau activities of YZT *in vitro* AD models, 7PA2 and SH-SY5Y-P301L cells, we found that YZT did not regulate the protein levels of full length APP (Fl-APP) and CTFs. These results indicate that YZT neither influences APP processing nor degrades APP metabolites. Since AD has both Aβ and tau related abnormalities, we tested the effect of YZT on the levels of tau in SH-SY5Y-P301L cells. Our results show that YZT reduced the levels of total tau in SH-SY5Y-P301L lysates. To confirm the tau-reducing activity of YZT observed in *in vitro* studies, we used P301S tau mice and 3XTg-AD transgenic mice for short-term and long-term *in vivo* studies. The P301S tau mice and 3XTg-AD mice were orally administered YZT (1, 2, or 4 g/kg/day). When we convert the dose used in a mouse to a human dose based on surface area for humans (Tao et al., 2013), 2 g/kg/d of YZT in mouse is equivalent to 0.16 g/kg in human. According to the Chinese pharmacopeia, the daily recommended dose of YZT for human is 0.026 g/kg. The dose used in our animal study is relatively 6 times higher than the dose typically prescribed for human. We used these doses because our cell tests showed no toxicity at this and greater levels; we wished to achieve significant brain levels in order to be able to fully assess the effects of YZT. Indeed, a recent study done elsewhere used even higher doses such as 4 or 8 g/kg/d to assess the *in vivo* effect of YZT with no toxic effects, (Xu et al., 2013). Since YZT components permeates the blood brain barrier (BBB) in acceptable concentrations (Tao et al., 2013) and we wished to achieve therapeutic effects in the brain, we utilized 1–4 g/kg/d of YZT, which is the dose that had previously used in a pharmacokinetic study to observe the brain permeable components of YZT (Reagan-Shaw et al., 2008).

A possible mechanism by which YZT decreases tau aggregation is by decreasing the phosphorylation of tau. In our experiments, YZT treatment decreased phosphorylated tau, which is the stimulator of tau aggregation (Biernat et al., 1992; Lewis et al., 2000). Although our results evidently demonstrated the capability of YZT to inhibit the advancement of tau aggregation, the underlying mechanism of YZT in reducing tau aggregation is to be disclosed. The p62/SQSTM1-inducing activity of YZT represents an intriguing and interesting finding. Multifunctional role of p62 was observed in several neurodegenerative entities such as tau hyperphosphorylation, association with tau protein and facilitates selective p62 mediated autophagy (Caccamo et al., 2017). Besides, p62 increasing effect of YZT, other antioxidants proteins such as HMOX1 and TXRD1 were also upregulated by YZT probably through the Nrf2-ARE signaling pathway. Upregulation of SLC7A11 in neuronal cells consider as to be the resistance to oxidative stress (Lewerenz et al., 2012). Similarly, HMOX1 also shows the resistance to oxidative stress facilitated cell death (Chen et al., 2000). Higher expression of PBX1, TXNRD1 and bHLHE22 gene shows that, which support neuronal growth, cell differentiation and maturation (Soerensen et al., 2008; Sgado et al., 2012; Dennis et al., 2019). The pathway analysis identified the modulation of ubiquitinoylation by targeting deubiquitylating enzyme (DUB), USP21, and its substrates as one of the pathways significantly changed amongst differentially expressed genes in response to YZT treatment (Figure 5). USP21 is a deubiquitinase, which promote deubiquitination process in protein degradation, deubiquitinating enzymes regulates centrosome regulation, chromosomal stability (Urbe et al., 2012). The study on reversible ubiquitination is progressing rapidly. USP21 of USP sub family, prominently regulates several pathway signals to interact with MEK2 and deubiquitinate MEK2 directly, thus promoting the tumor growth in cells inducing stabilization of MEK2 by activating ERK1/2 pathway (Li et al., 2018). In spite, its actual performance in tau stabilization and other proteins involved in neurodegeneration are yet to be established. It is imperative to initiate further studies on this subject to elucidate its participatory role in eradicating neurodegenerative protein. One interesting finding is that the expression of genes nitric oxide synthase one adaptor protein (NOS1AP) also recognized as carboxyl-terminal PDZ ligand of neuronal nitric oxide synthase protein (CAPON); 2) Repressor element-1 binding transcription factor (REST) are involved in tau aggregation and cytoplasmic aggregates formation respectively, were strongly downregulated by YZT (Figure 5). CAPON has recently shown as a molecular linker that connects Aβ amyloidosis and tau pathology (Hashimoto et al., 2019). Increase of CAPON was observed in the APP mutant knock in (KI) mice brain. Overexpression of CAPON in APP KI mice increased tau phosphorylation in mice whereas the reduction of CAPON decreased tau pathology and neuronal loss in P301S tau transgenic mice. However, CAPON-induced cell death was shown to be only attributed to a Aβ-dependent mechanism but not tau (Hashimoto et al., 2019). Ubiquitin-based proteasomal degradation is induced by REST during the neuronal differentiation (Ballas et al., 2005). REST is dormant in mature neurons, however REST can be stimulated by the vulnerable hippocampal neurons in disease like ischemia (Calderone et al., 2003; Formisano et al., 2007) and epileptic seizures (Palm et al., 1998), and also found in Huntington disease (Zuccato et al., 2003; Zuccato et al., 2007).

This study also predicted YZT as an activator of SQSTM1, could be as an antioxidant, and if true, then microarray gene expression profiling could be combined with CMap (Clue) to reveal mechanisms of actions and to identify new health benefits of YZT. In addition, YZT also had connections with aromatase inhibitors (99.27), including exemestane and androsta-1,4-dien-3,17-dione, and FXR antagonist (97.46), including guggulsterone and 15-deoxy-delta(12,14)- prostaglandin J2 (Figure 5). These predictions warrant further characterization. Moreover, it has been shown that the key markers of YZT such as protopine, allocryptopine, tetrahydropalmatine, tetrahydroberberine, corydaline, palmatine, dehydrocorydaline and imperatorin are brain permeable (Tao et al., 2013) , which might explicate the tau-reducing effect of YZT in the central nerveous system of transgenic AD mice; key markers permeable in brain crossing the blood brain barrier namely. Although YZT components reach the brain, yet it is possible that YZT also exerts therapeutic effects in other areas of the body. Since YZT is encompassed of numerous small molecules, distinguishing the effective compounds and knowing their mechanism of action in disease modification will involve further work.

**FIGURE 8:**
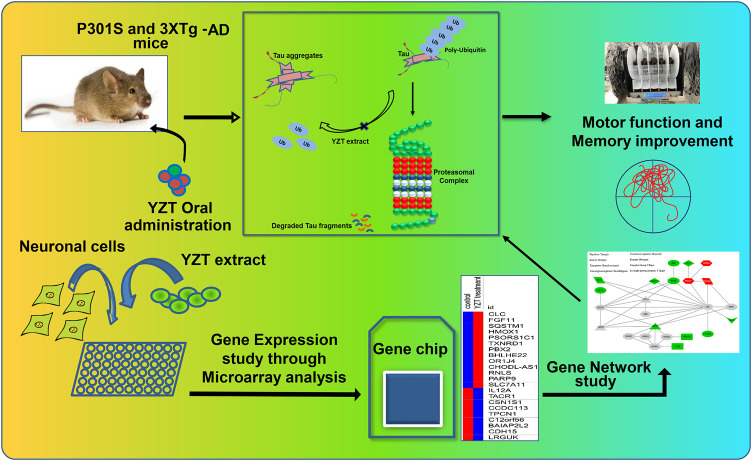
Schematic illustration of YZT's effect on tau pathology and its mechanism of action.

In conclusion, based on experimental results, we found that YZT had remarkable tau-reducing and cognitive-enhancing activities (Figure 8). Based on the tau-reducing activity, the motor- and memory- enhancing properties and anti-oxidative capacity of YZT, results suggest that YZT can be established as a health supplement or may possibly distributed as a raw material for prescriptions to prevent or cure AD. Present study establishes not only valuable evidence about the disease modifying function of YZT, but also provides a strategy to create TCM into the network pharmacology studies.

## Data Availability Statement

The raw data supporting the conclusions of this manuscript will be made available by the authors, without undue reservation, to any qualified researcher.

## Ethics Statement

All animal experiments were approved by the Hong Kong Baptist University Committee on the Use of Human and Animal Subjects in Teaching and Research (HASC approval # HASC/13-14/0165) and by the Committee on the Use of Live Animals for Teaching and Research (CULATR #3314), at the University of Hong Kong. All animal experiments were performed in accordance with the relevant guidelines and regulations of both HASC and CULATR.

## Author Contributions

Conceptualization: ML, SSKD, CH. Methodology: AI, SK, YL, AK, SS, SM, BT, SSKD. Investigation: AI, SK, YL, AK, SS, SM. Data curation: SSKD, AI, SK, YL, AK, ML, JS, JL. Writing original draft: AI, SK, YL, SSKD, CH, BT. Writing review and editing: ML, JS, JL,SSKD, CH, JT, and KC. Funding acquisition: ML, SSKD. Resources: ML, SSKD.

## Funding

This study was supported by the grants of Hong Kong Health and Medical Research Fund (HMRF 12132061, HMRF 14150811, HMRF 15163481, HMRF 17182541, HMRF 17182551), the General Research Fund from Hong Kong Government SAR (GRF/HKBU 12101417, GRF/HKBU 12100618), and research grant from the Hong Kong Baptist University (HKBU/RC-IRCs/17–18/03). This study also supported by the National Natural Science Foundation, China (NSFC 81773926, NSFC 81703487) and Shenzhen Science and Technology Innovation Commission, China (JCYJ20180507184656626, JCYJ20180302174028790).

## Conflict of Interest

The authors declare that the research was conducted in the absence of any commercial or financial relationships that could be construed as a potential conflict of interest.
