# Supplementary figures and images for: Yuan-Hu Zhi Tong Prescription Mitigates Tau Pathology and Alleviates Memory Deficiency in the Preclinical Models of Alzheimer’s Disease
Source: Front Pharmacol. 2020 Oct 30;11:584770. doi: 10.3389/fphar.2020.584770 (PMC7663173; doi:10.3389/fphar.2020.584770)

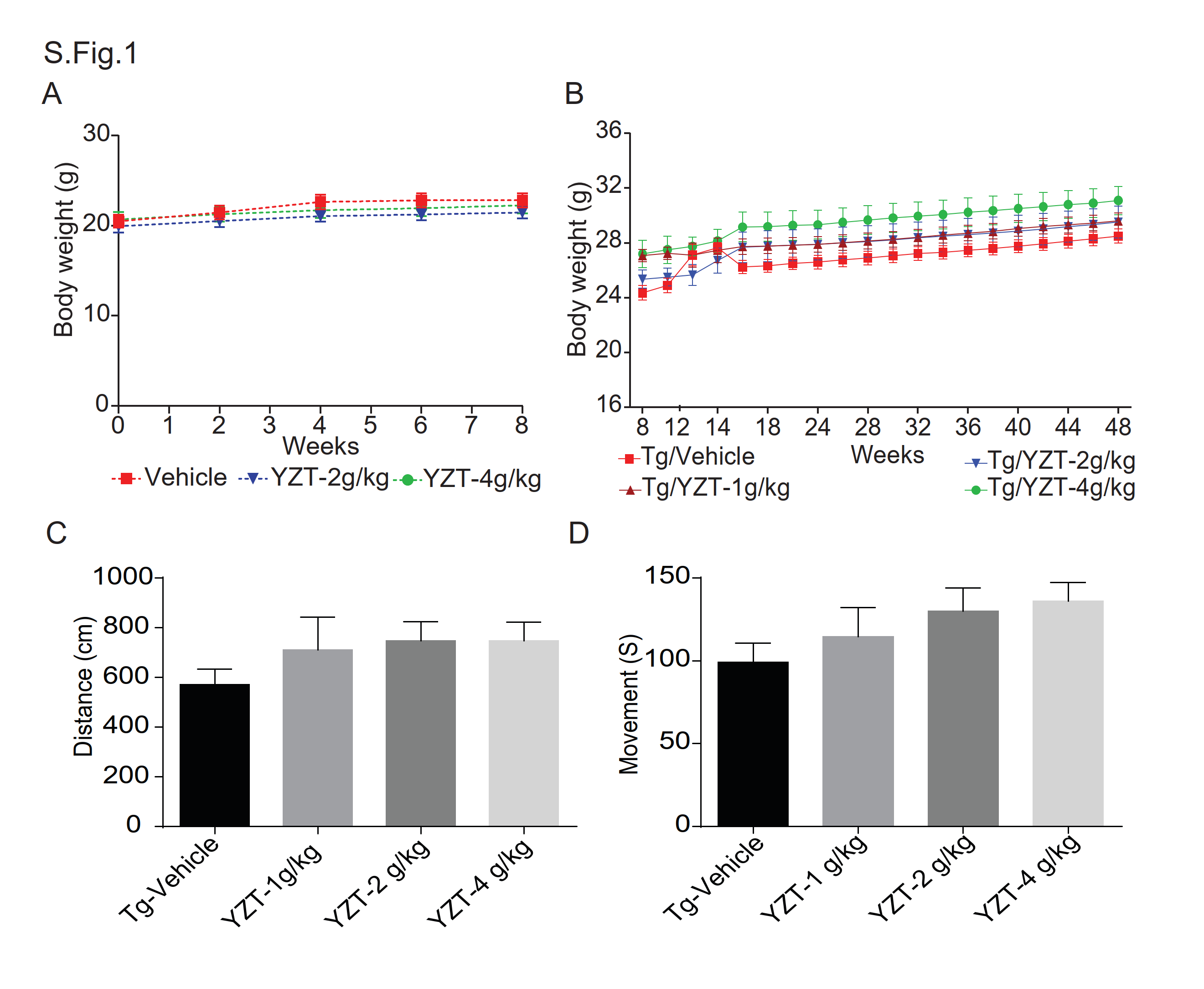

Supplement: Supplementary file 1 [file image1.tif]

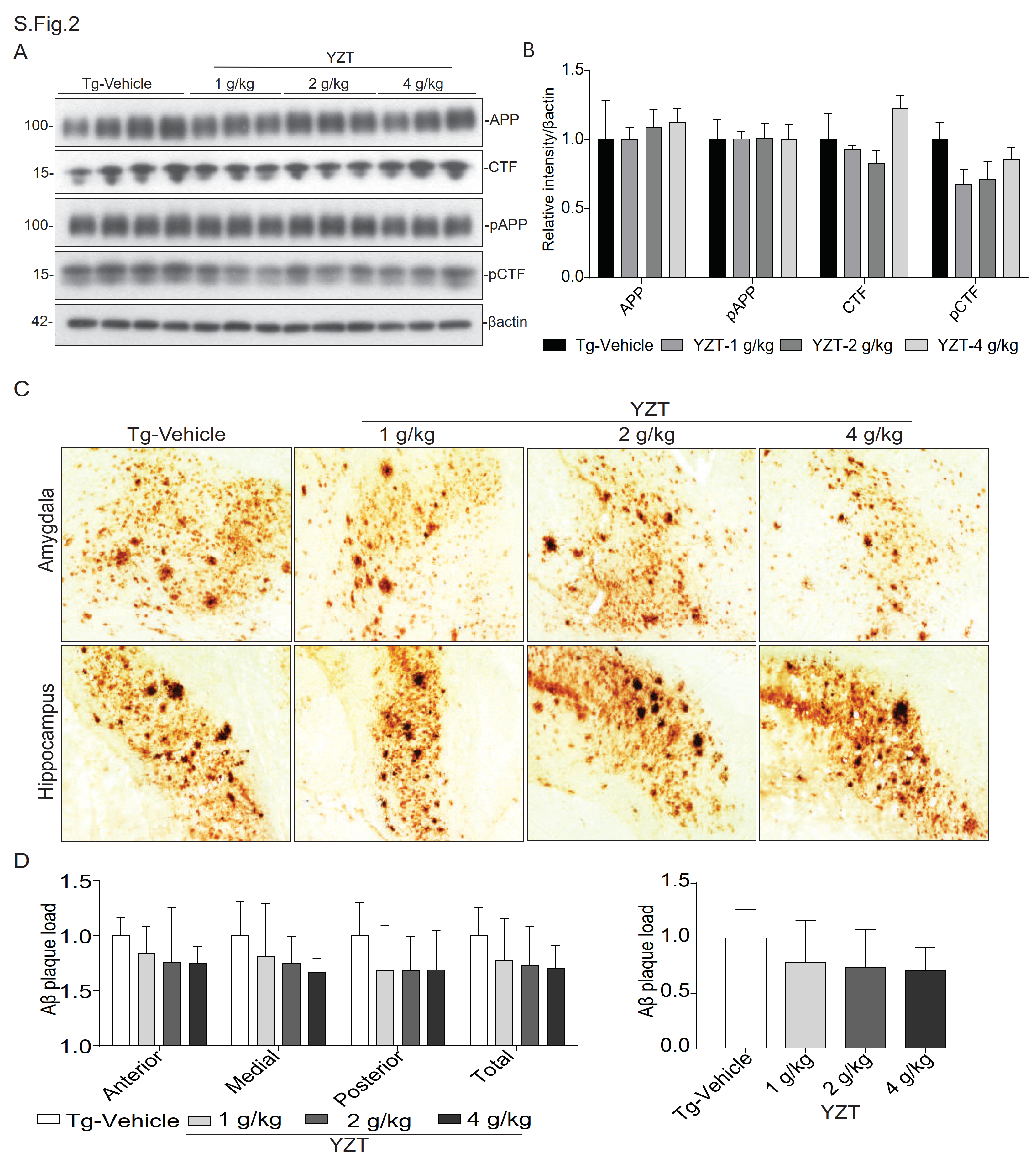

Supplement: Supplementary file 2 [file image2.tif]

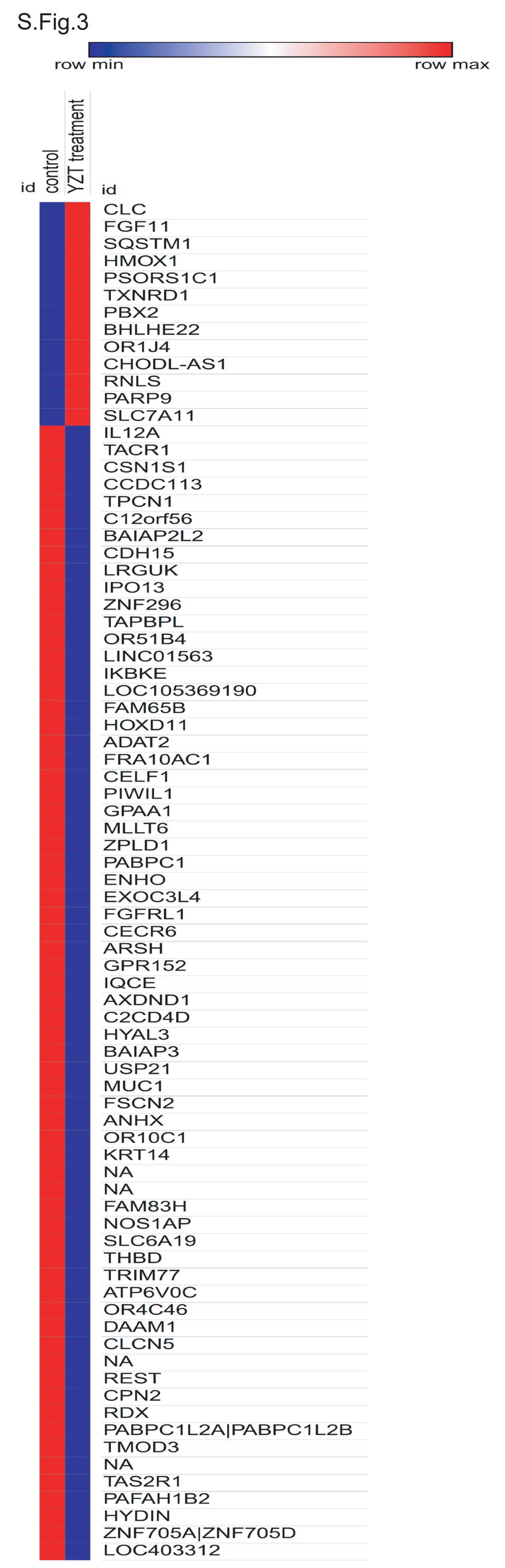

Supplement: Supplementary file 3 [file image3.tif]

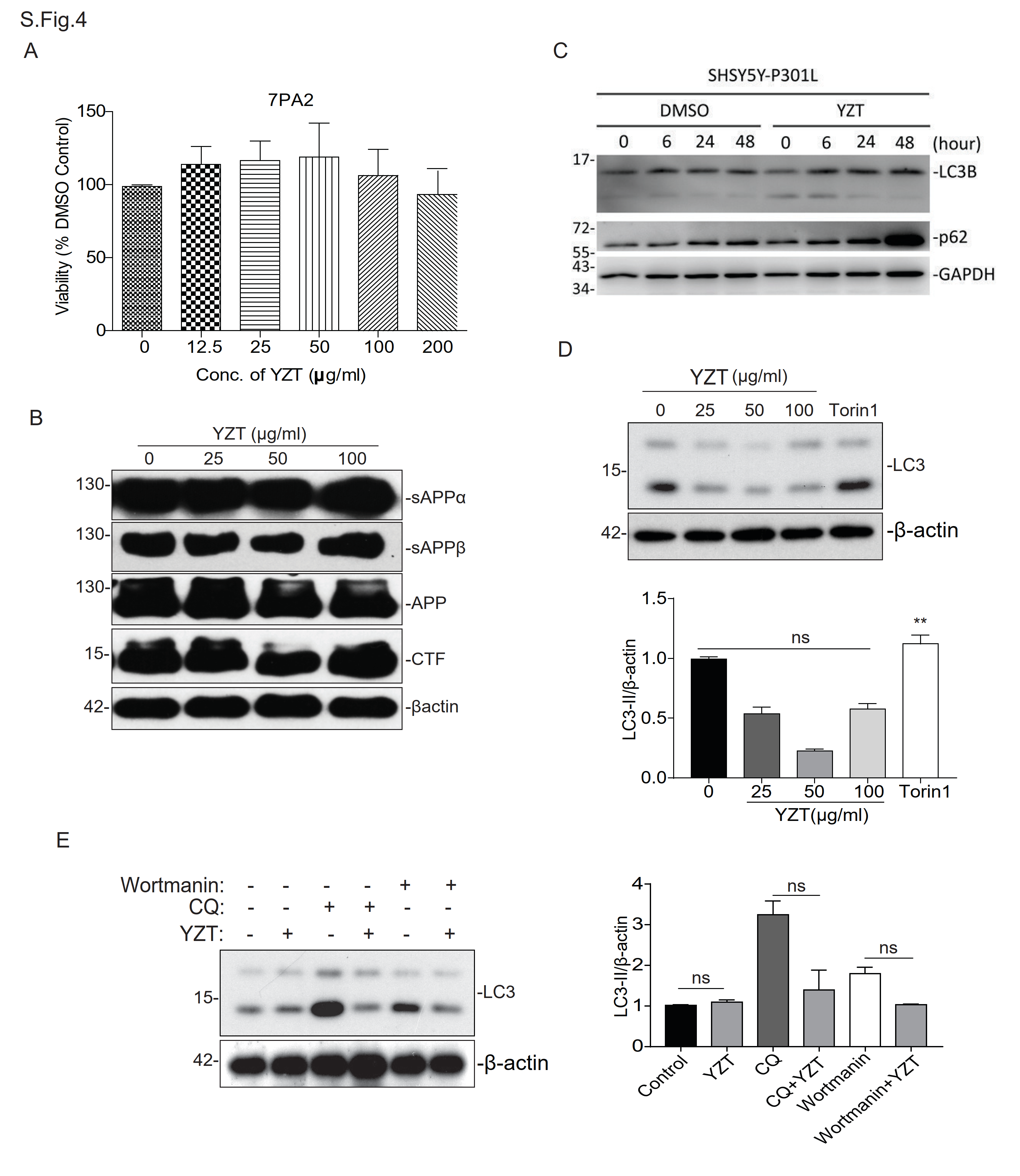

Supplement: Supplementary file 4 [file image4.tif]

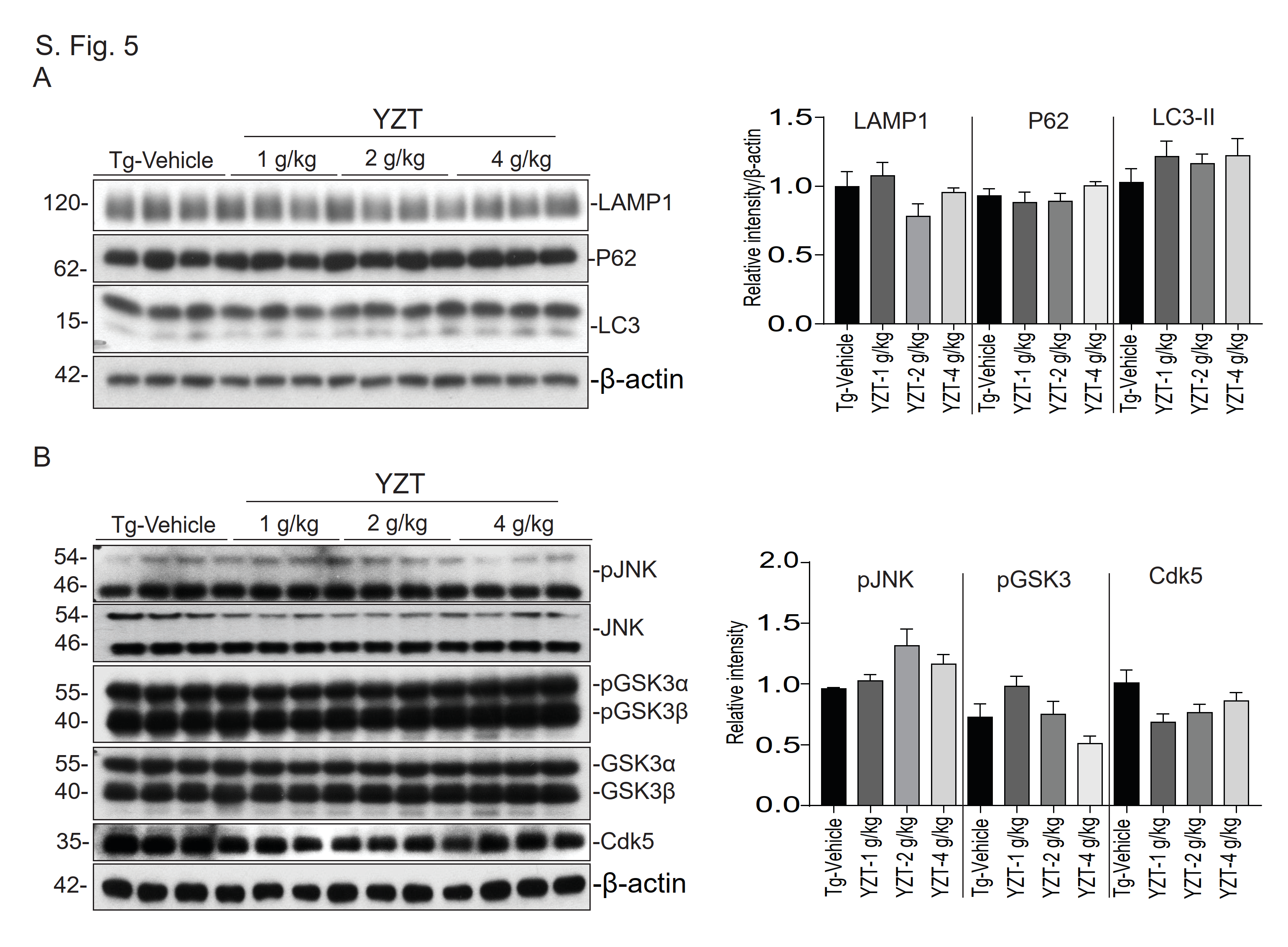

Supplement: Supplementary file 5 [file image5.tif]
